# An analysis of fellowship training of kidney transplant surgeons in a Brazilian state

**DOI:** 10.1590/2175-8239-JBN-2024-0056en

**Published:** 2024-10-18

**Authors:** Salim Anderson Khouri Ferreira, João Henrique Sendrete de Pinho, Juliano Offerni, Helady Sanders-Pinheiro

**Affiliations:** 1 Universidade Federal de Juiz de Fora, Hospital Universitário, Serviço de Transplante Renal, Juiz de Fora, MG, Brazil.; 2 Universidade Federal de Juiz de Fora, Núcleo Interdisciplinar de Estudos e Pesquisas em Nefrologia (NIEPEN), Juiz de Fora, MG, Brazil.; 3 University of Manitoba, Faculty of Health Science, Department of Surgery, Urology, Kidney Transplant, Rady Winnipeg, Canada.

**Keywords:** Kidney Transplantation, Surgeons, Professional Training, Career Choice, Medical Education, Graduate, Health Services Accessibility

## Abstract

**Introduction:**

The lack of specialized professionals potentially contributes to the inability to meet the demand for kidney transplantations. Moreover, there is no universal proposal for the training process of transplantation surgeons. We aimed to explore the characteristics of the training program and professional activities of kidney transplantation teams in the state of Minas Gerais, Brazil.

**Methods:**

We invited the surgeons of all 19 active kidney transplantation centers in Minas Gerais to participate in this cross-sectional study. Demographic and professional training data were compared using linear and logistic regression models.

**Results:**

The response rate among the centers was high (89%); half of the surgeons answered the survey (39/78). Most of the centers were public teaching institutions, under a production-based payment contract, with a mean of 6 ± 2.4 surgeons/team; 94.2% of the centers had urologists. The surgeons were 95% male (age of 46.3 ± 9.7 years) and 59% were urologists. Most were involved in organ procurement and transplantation; only one surgeon worked exclusively with transplantation. The mean period since training was 13 ± 9.4 years, with a mean of 10 ± 9.7 years as part of the transplantation team. Only 25.6% had specialized or formal training in transplantation, with only one completing a formal medical residency for kidney transplantation. The lack of training programs was the most frequently cited reason.

**Conclusion:**

Kidney transplantation surgeons are not exclusive and most have not completed a formal fellowship program in transplantation because they are not available. These data indicate the need to improve training programs and facilitate the formation of new kidney transplantation teams.

## Introduction

Kidney transplantation (KT) is the treatment of choice for most patients with chronic kidney disease, leading to longer survival and a better quality of life than dialysis treatment^1^. Brazil has the fourth largest absolute number of KT, which has been increasing annually, with 6,047 procedures performed in 2023^2^. Nevertheless, the waiting list continues to grow as we can only meet approximately 40–50% of the estimated need^2,3^. The lack of steady increase in organ donation rate contributes to the imbalance of the system, which may be tracked by the high rates of family refusal to donate and the low rates of brain death notifications^2,3,4,5^. Other difficulties are issues directly related to the operational aspects of transplant teams, such as economic and social disparities between states and regions and financial and structural limitations of some transplant programs^5,6,7 8,9^.

KT is dependent on a specialized, complex, and coordinated set of actions, which involves many levels of the healthcare system. The final step in this process is the KT surgical procedure, performed by skilled and trained professionals. According to the Global Observatory on Donation and Transplantation (GODT), more than 102,090 KTs were performed worldwide in 2022^10^. However, there is no universal and well-defined proposal for the training process of KT-specialized surgeons. The content, time, and minimum curriculum vary across countries, as does the need for accreditation^11,12,13,14^. Following strict safety regulations, healthcare systems have defined rules and requirements for formation of KT teams^11,15^.

In Brazil, there are very few specialized KT training programs, including formal medical residency or specialization programs. There are only 11 programs available (Medical Residence National Committee, personal communication by e-mail, 2021). However, similar to other countries, accreditation as a KT team in our public health system follows strictly defined rules and requirements. To be part of a KT team, surgeons must be urologists or general surgeons who had completed a specific medical residency program or a *latto sensu* postgraduate course (specialization program) and must have professionally practiced in an accredited KT center for a minimum of 6 months^16^. The same criteria are applied to organ procurement accreditation^16^. The Brazilian legislation dates back to 1997, and updates have been published^16^. Since then, specific training programs have been created at a lower rate than what the demand appears to be. Another relevant aspect is the practice pattern of these very specialized surgeons. The long time required for the transplant procedure, the requirements for high-level hospitals, and an often limited number of procedures, make KT specialization not very attractive for young surgeons. Based on this, one can speculate that the shortage of specialized KT surgeons could be a potential reason for the inappropriate number of KT performed in our country.

To our knowledge, there is only one study on the demographic and practice characteristics of KT surgical teams in Brazil, but aspects of the training of these surgeons was not explored^17^. Therefore, the present study aimed to evaluate the characteristics of KT teams in Minas Gerais, the state with the second-highest number of KTs in Brazil, exploring training and practice characteristics.

## Methods

We invited all active KT centers in Minas Gerais at the time of the conception of the study (2021)^3^ to participate in this cross-sectional study. We considered as active centers those that had performed at least one KT/year in the last 5 years^3^. The invitation to participate and the data collection form were sent by email to the heads of the active centers, as registered at the Brazilian Transplant Register^3^. After acceptance, the invitation and data collection forms were sent to all KT team members listed by the KT coordinator. Before answering the questions, participants agreed to participate by electronically signing an informed consent form.

The study was conducted in accordance with the Declaration of Helsinki and its amendments and was approved by the Research Ethics Committee of the University Hospital of the Federal University of Juiz de Fora (registry: CAAE48030521.9.0000.5133; approval number:4.825.883).

We collected demographic data and data on the characteristics of the transplant centers, team compositions, forms of remuneration, and professional training of the surgeons of each team.

The following data were collected for centers: location (capital or other); type of hospital (teaching institution or other); specialist composition of the transplant teams (urologist, vascular surgeon, general surgeon, or other); number of exclusive specialists in the KT team; activity in organ donation process; pediatric transplantation; percentage of transplants paid by the Sistema Único de Saúde (SUS), the Brazilian Public Health System (100%, 75–100%, 50–75%, 25–50%, less than 25%, and 0%); team remuneration (fixed monthly salary, per-production, or otherwise); remuneration for long-distance availability; and team contract with the institution (group contract or formal public contract)^12,17^.

For the KT surgeons, we collected data on age, sex, and race. Regarding professional training, we evaluated the time since medical school graduation (years), surgical specialty (urology, vascular surgery, or general surgery), type of postgraduate training in transplantation (residency or specialization), time elapsed since training in transplantation (years), time as part of the KT team (years), type of activity in the KT field (transplantation and kidney donation), and participation in more than one KT team^12,17^.

Descriptive data were reported as mean (± standard deviation), median (minimum and maximum), and proportion according to the type of variable. The normality of continuous variables was assessed using the Kolmogorov-Smirnov test, and comparisons were performed using Student’s *t*-test or Mann*-*Whitney U test, according to the distribution of the variables. Categorical variables were compared using the chi-square test or Fisher’s exact test. To compare KT surgeons according to specific training levels, we evaluated the association between exploratory variables using linear and logistic regression models for quantitative and categorical variables. Statistical significance was set at p < 0.05, and all data were analyzed using Stata (Version 14, StataCorp, College Station, TX, USA).

## Results

**Figure 1:**
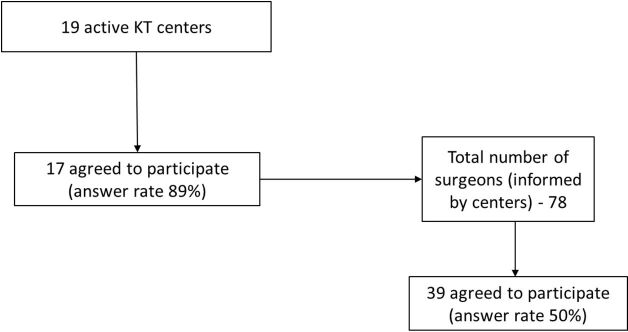
Sample flow. KT – kidney transplantation.

Of the 19 active KT centers in Minas Gerais, 17 (89%) agreed to participate and 50% of the surgeons, informed by the center’s representative, answered the survey (Figure 1). Forty-seven percent were located in the capital, 88% were teaching institutions, and in 94% the SUS payed more than 75% of the KTs performed. The number of surgeons was 6 ± 2.4 / team, composed mainly of urologists (3.5 ± 1.9 / team). Regarding the forms of payment, 76.5% of the surgeons received payment based on production and 76.5% did not have formal working contracts with the hospital (Table 1).

**Table 1:** Characteristics of the 17 active KT centers studied

Variable	% / Mean ± SD
Located in the capital	47% (8/17)
Teaching hospital	89% (15/17)
Number of surgeons in the team	6 ± 2.4
Urologist	3.47 ± 1.94
Vascular surgeon	1.65 ± 1.66
General surgeon	0 (0–2)*
Composition of the KT team	
Urologist	94.2% (16/17)
Vascular surgeon	64.7% (11/17)
General surgeon	29.4% (5/17)
Transplant surgeon	17.6% (3/17)
Exclusively involved in KT	5.9% (1/17)
Organ recovery	94.1% (16/17)
Number of surgeons in organ recovery	3.65 ± 2.26
Pediatric transplantation	47% (8/17)
Payment by Unified Health System	
100%	70.6% (12/17)
75% or more	23.5% (4/17)
Less than 25%	5.9% (1/17)
Payment by production	76.5% (13/17)
Payment for distance call	17.6% (3/17)
Working contracts	
Group of surgeons	76.5% (13/17)
Formal public	23.5% (4/17)

KT: kidney transplantation, SD: standard deviation.

*median and interquartile range.

**Figure 2:**
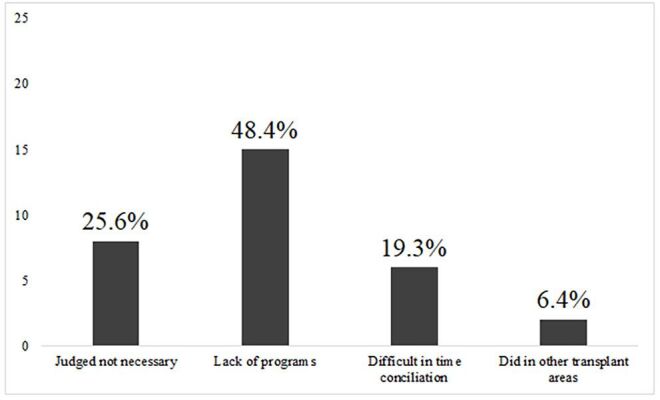
Reasons for lack of specific kidney transplantation training.

KT surgeons were mostly male (95%) with a mean age of 46.3 ± 9.7 years. Most surgeons performed their medical residency in surgical areas, the mean time since training in KT was 13 ± 9.4 years, and the mean time working in KT teams was 10 ± 9.7 years. Most of the KT surgeons were involved in both KT and organ donation. Only one surgeon worked exclusively in KT field, and 13% of the surgeons were part of more than one team. Only 25.6% of the participants had completed residency or specialization in transplantation, and only one had completed a formal residency in KT (Table 2). Among the surgeons who did not undergo specialization or medical residency in KT, the most frequently cited reasons were lack of programs (48%), judged as not necessary (26%), and difficult to balance with work (19%) (Figure 2). Surgeons with specific training in transplantation showed similar demographic and practice characteristics, except that they were more likely to receive production-based payments because they did not have formal contracts with hospitals (Table 3).

**Table 2:** Demographic, education, and practice characteristics of surgeons working in KT

Variable	% / Mean ± SD
Sex (male)	95% (37/39)
Caucasian	92.3% (36/39)
Age (years)	46.3 ± 9.7
Time since graduation (years)	21.6 ± 10
Surgery specialization	
Urology	59% (23/39)
General surgery	20.5% (08/39)
Vascular surgery	17.9% (07/39)
Kidney transplantation	2.6% (01/39)
Type of specialization program	
Medical residence in specialized area	74.4% (29/39)
Fellowship in transplant surgery	20.5% (08/39)
Fellowship in KT surgery	2.6% (01/39)
Medical residence in KT surgery	2.6% (01/39)
Specialization or residency in transplant or KT	25.6% (10/39)
Time since training in transplantation (years)	13 ± 9.4
Time acting in KT teams (years)	10 ± 9.7
Areas of activity in KT teams	
Transplants and organ recovery	69.2% (27/39)
Transplant only	20.5% (08/39)
Organ recovery only	10.3% (04/39)
Participation in more than one team	13% (05/39)
Exclusively involved in KT	0

KT: kidney transplantation, SD: standard deviation.

**Table 3:** Comparisons between demographic and practice characteristics of surgeons with and without specific training in transplantation or KT.

Variable	With specific training (n = 10)	Without specific training (n = 29)	OR (95% CI), p-value
***Demographic and formation***		
Age (years)	44.2 ± 10.9	46.9 ± 9.4	1.03 (0.95–1.12), p = 0.756
Male sex	90% (9/10)	96.5% (28/29)	2.8 (0.16–49.21), p = 0.418
Caucasian	90% (9/10)	93.1% (27/29)	1.22 (0.36–4.09), p = 0.166
Time since medical school (years)	19.8 ± 11.2	22.2 ± 18.6	1.03 (0.95–1.11), p = 0.710
Time since KT training (years)	13 ± 9.13	13 ± 9.72	1.00 (0.93–1.08), p = 0.504
***Professional profile***		
Teaching hospital	90% (9/10)	96.5% (28/29)	0.34 (0.02–6.81), p = 0.418
Time in KT (years)	8.5 ± 6.75	10.5 ± 10.6	1.04 (0.95–1.13), p = 0.757
Number of surgeons on the team	5.9 ± 2.4	7.1 ± 2.3	1.24 (0.91–1.68), p = 0.898
Performs KT and organ donation	80% (8/10)	65% (19/29)	1.46 (0.44–4.82), p = 0.622
Number of KT teams	1.2 ± 0.42	1.1 ± 0.31	0.52 (0.07–3.86), p = 0.259
Team paid 100% by SUS	40% (4/10)	69% (20/29)	0.30 (0.07–1.38), p = 0.104
Payment by production	90% (9/10)	48.3% (14/29)	6.66 (1.72–25), p = 0.021
Formal work contract	10% (1/10)	51.7% (15/29)	0.15 (0.04–0.58), p = 0.021

KT: kidney transplantation, SD: standard deviation, OR: odds ratio, 95% CI: 95% confidence interval, SUS: Sistema Único de Saúde.

## Discussion

In this analysis conducted in Minas Gerais, the majority of the 17 KT centers were located in teaching hospitals, most KTs were paid for by the Brazilian Public Health System, and most professionals were paid by production and did not have formal contracts. The 39 evaluated surgeons were mostly male with more than 20 years since graduation, most were urologists, and without specific training in KT (residency or specialization) due to lack of opportunity or availability. This was a population-based cross-sectional study that, for the first time to our knowledge, evaluated the training and activity characteristics of surgeons performing KTs in Brazil. Although our sample had characteristics specific to Brazil, Brazil is one of the countries with high transplant activity, and our challenges could be similar to those of other countries that share these characteristics.

We found that the centers were predominantly in the capital and in teaching hospitals, revealing the complexity of KT as a procedure. Similar findings were reported by Ximenes et al.^17^ in the only other Brazilian study that evaluated 20 teams acting in the state of São Paulo, the state with the highest number of KTs in the country^2,3^. The distribution of KT centers in areas with better economic resources^9,18^ and highly structured facilities, such as teaching hospitals, has been previously described^19^. The main payer for KT procedures was the public health system, which increases access to treatment, a characteristic of countries with a high prevalence of KT ^18,20^. We found a higher percentage of publicly financed procedures (more than 75% of procedures) when compared with the other Brazilian study, 94% vs. 55%^16^. On the other hand, remuneration for long-distance availability is higher in São Paulo^17^. However, regardless of these small differences, the work scenario is far from ideal, primarily because of the fragility of working conditions in temporary contracts.

More than 90% of KT surgeons were male, similar to previous reports^21,22^. For the first time, the details of the professional training of Brazilian KT surgeons were explored. Most professionals were trained in KT during their surgical residency, but only one-quarter underwent specific training in KT through a medical residency or fellowship program. This percentage is lower than that reported by Florence et al.^22^ in a sample from the United States (US). They studied 171 surgeons working in KT and reported that 95% had completed fellowship training in transplantation. This difference is also reflected in the total training time, which was 2.6 years shorter in our sample^22^. Currently, after legislation with detailed norms and educational content was established, the US has 66 transplant training programs, most of multiorgan and 205 KT programs^23,24^. In some countries, such as Germany, there is no regulation for medical training in organ transplantation, and there is no need for specialization certification in the field to practice solid organ transplantation. Certification from medical association in the discipline of surgery has been proposed^11^. Two German studies reported low levels of specific training in transplantation, with only 17% and 11.8% of transplant surgeons having any type of certification^10,13^.

Currently, in Brazil, a universal and well-defined proposal for KT training is lacking, even though there are accredited specialized training programs for KT. The Brazilian regulations for the composition of KT teams for accreditation by the Ministry of Health^16^ stipulate that the team must have two urologists or one urologist and one general surgeon with residency or a specialist title, at least six months of formal training in KT in a teaching or excellence hospital in the field^16^. However, unlike US^12^, in Brazil there are no details on how the training program should be implemented, and, for example, there is no minimum number of required procedures assisted or performed under supervision^25^. Some countries have defined the minimum number of procedures and proficiency examinations as a requirement for accreditation as part of KT surgery teams^10,26^. After the creation of the Brazilian regulations for medical residence in transplantation in 2010^25^, there was a growth in medical residency programs in KT and specializations in the country. Currently, there are 11 centers with surgical training exclusively in KT, nine in urology, and two in vascular surgery (personal communication, Brazilian National Committee of Medical Residency, 2020). Thus, the current paths for KT training in Brazil are a specific medical residency program, a *latto sensu* postgraduate course (specialization program), or the professional practice of a specialist in urology or general surgery in a KT-accredited center. Although the number of specialized programs is growing, most of the KT surgeons in our study reported the unavailability of training programs as the reason for not completing this kind of complementary and necessary education. Information about the fulfillment of entry-level positions in this KT-specialized program is not available.

The mean time of work in KT teams was shorter in our survey than that reported by the other Brazilian study, possibly because the KT career is new, highly specialized, and less attractive, as reported^14,27^. One major finding of our study was that surgeons with specific training in transplantation had less favorable institutional contracts and forms of remuneration. This may be because specific training in KT was only recently made available; therefore, surgeons who performed this training are younger and still at the start of their careers. Another relevant aspect is that even with the most appropriate training, the work field still does not favor these professionals since specific training in KT is not required by regulations^12,13^.

Currently, transplant surgery in not recognized as a specialty by the Federal Council of Medicine and the Associação Médica Brasileira (AMB), the Brazilian Medical Association^28^. This makes the selection process for this type of professional difficult, as the legislation does not allow for the opening of positions for specialties not included in the AMB list. Consequently, it is difficult to form new teams or expand active KT teams in public entities such as many Brazilian KT centers^2^. The low rate of professionals with specific training in KT in the centers and the more precarious forms of remuneration and contracts that we found reflect the difficulties mentioned above and reinforce the impact of the lack of specific regulations.

This study had some limitations, mainly because it was a survey-based study. Although the response rate of the heads of the KT centers in Minas Gerais was high (89%), the response rate of the surgeons from participating centers was 50%, which may have biased the results. However, the percentage of participation was similar to previous reports^12,22^. Even though our study was based on data from one Brazilian state, it provides an initial draft of the actions needed to improve KT training and highlights the need for more detailed national data. Another aspect that needs further exploration is the potential improvement in performance in centers with professionals with specific training in KT as part of their teams.

In conclusion, we found that the surgeons who worked in KT centers in Minas Gerais were mostly males, urologists, involved in organ procurement and transplantation, and without specific training in KT due to lack of opportunity or availability. Most KT centers were teaching institutions that paid per production and by the public health system. A minority of the surgeons had a residency or fellowship in transplantation and worked under less favorable contracts. We believe that these data could help medical associations and government institutions to design policies to increase the number of specialized KT surgeons and skilled teams to ultimately increase the number of KTs.

## Data Availability

The datasets generated and/or analyzed during the current study are available from the corresponding author upon request.
