# Supplementary material for: An analysis of fellowship training of kidney transplant surgeons in a Brazilian state
Source: J Bras Nefrol. 2024 Oct 18;46(4):e20240056. doi: 10.1590/2175-8239-JBN-2024-0056en (PMC11493301; doi:10.1590/2175-8239-JBN-2024-0056en)
Supplement: Translation

# Translation: Uma análise do treinamento de cirurgiões de transplante renal em um estado brasileiro

**DOI:** 10.1590/2175-8239-JBN-2024-0056pt

**Authors:** Salim Anderson Khouri Ferreira, João Henrique Sendrete de Pinho, Juliano Offerni, Helady Sanders-Pinheiro

**Affiliations:** 1 Universidade Federal de Juiz de Fora, Hospital Universitário, Serviço de Transplante Renal, Juiz de Fora, MG, Brasil.; 2 Universidade Federal de Juiz de Fora, Núcleo Interdisciplinar de Estudos e Pesquisas em Nefrologia (NIEPEN), Juiz de Fora, MG, Brasil.; 3 University of Manitoba, Faculty of Health Science, Department of Surgery, Urology, Kidney Transplant, Rady Winnipeg, Canada.

**Keywords:** Transplante de Rim, Cirurgiões, Capacitação Profissional, Escolha da Profissão, Educação de Pós-Graduação em Medicina, Acessibilidade aos Serviços de Saúde

## Abstract

**Introdução:**

A falta de profissionais especializados pode contribuir para incapacidade de atender à demanda por transplantes renais. Além disto, não existe uma proposta universal para o processo de formação de cirurgiões transplantadores. Nosso objetivo foi explorar características do programa de treinamento e atividades profissionais das equipes de transplante renal no estado de Minas Gerais, Brasil.

**Métodos:**

Convidamos os cirurgiões dos 19 centros de transplante renal ativos em Minas Gerais a participarem desse estudo transversal. Comparamos dados demográficos e de formação profissional utilizando modelos de regressão linear e logística.

**Resultados:**

A taxa de resposta entre os centros foi elevada (89%); metade dos cirurgiões responderam à pesquisa (39/78). A maioria dos centros eram instituições públicas de ensino, com contrato de remuneração por produção, com média de 6 ± 2,4 cirurgiões/equipe; 94,2% dos centros tinham urologistas. Os cirurgiões eram 95% homens (idade entre 46, 3 ± 9,7 anos) e 59% eram urologistas. A maioria estava envolvida em captação e transplante de órgãos; somente um trabalhava exclusivamente com transplante. O período médio desde a formação foi de 13 ± 9,4 anos, com média de 10 ± 9,7 anos como parte da equipe de transplante. Apenas 25,6% possuíam formação especializada ou formal em transplante, com somente um tendo concluído residência médica formal em transplante renal. A falta de programas de treinamento foi o motivo mais citado.

**Conclusão:**

Cirurgiões de transplante renal não são exclusivos e a maioria não concluiu um programa formal de treinamento em transplante pela falta de disponibilidade. Esses dados indicam necessidade de aprimorar programas de treinamento e facilitar a formação de novas equipes de transplante renal.

## Introdução

O transplante renal (TxR) é o tratamento de escolha para a maioria dos pacientes com doença renal crônica, proporcionando uma sobrevida mais longa e melhor qualidade de vida em comparação com o tratamento dialítico^1^. O Brasil é o quarto país no número absoluto de casos de TxR, com um aumento anual no número de procedimentos, totalizando 6.047 transplantes realizados em 2023^2^. No entanto, a lista de espera continua a crescer, pois conseguimos atender somente cerca de 40 a 50% da necessidade estimada^2,3^. A falta de um aumento constante na taxa de doação de órgãos contribui para o desequilíbrio do sistema, que pode ser detectado pelas elevadas taxas de recusa familiar em doar e pelas baixas taxas de notificações de morte encefálica^2,3,4,5^. Outras dificuldades são as questões diretamente relacionadas aos aspectos operacionais das equipes de transplante, como disparidades econômicas e sociais entre estados e regiões, além de limitações financeiras e estruturais de alguns programas de transplante^5,6,7 8,9^.

O TxR depende de um conjunto especializado, complexo e coordenado de ações, que envolve diversos níveis do sistema de saúde. A etapa final desse processo é o procedimento cirúrgico de TxR, realizado por profissionais capacitados e treinados. De acordo com o *Global Observatory on Donation and Transplantation* (GODT), mais de 102.090 TxR foram realizados mundialmente em 2022^10^. Entretanto, não existe uma proposta universal e bem definida para o processo de treinamento de cirurgiões especializados em TxR. O conteúdo, a duração e o currículo mínimo variam de acordo com o país, assim como a necessidade de acreditação^11,12,13,14^. Seguindo normas de segurança rigorosas, os sistemas de saúde definiram regras e requisitos para a formação de equipes de TxR^11,15^.

No Brasil, há um número muito pequeno de programas especializados de formação em TxR, incluindo programas formais de residência médica ou especialização. Existem somente 11 programas disponíveis (Comissão Nacional de Residência Médica, comunicação pessoal por e-mail, 2021). No entanto, semelhante a outros países, a acreditação como equipe de TxR em nosso sistema de saúde pública segue regras e requisitos estritamente definidos. Para integrar uma equipe de TxR, os cirurgiões devem ser urologistas ou cirurgiões gerais que tenham concluído um programa específico de residência médica ou um curso de pós-graduação *latto sensu* (programa de especialização) e devem ter atuado profissionalmente em um centro de TxR acreditado por um período mínimo de 6 meses^16^. Os mesmos critérios são aplicados à acreditação para captação de órgãos^16^. A legislação brasileira data de 1997, com atualizações publicadas posteriormente^16^. Desde então, os programas de treinamento específicos foram criados a uma taxa inferior à demanda aparente. Outro aspecto relevante é o padrão de prática desses cirurgiões altamente especializados. O longo tempo necessário para o procedimento de transplante, as exigências para hospitais de alto nível e um número frequentemente limitado de procedimentos tornam a especialização em TxR pouco atraente para cirurgiões jovens. Com base nisso, pode-se especular que a escassez de cirurgiões especializados em TxR pode ser uma possível razão para o número inadequado de TxR realizados em nosso país.

Até onde temos conhecimento, existe somente um estudo sobre as características demográficas e práticas das equipes cirúrgicas de TxR no Brasil, mas os aspectos da formação desses cirurgiões não foram explorados^17^. Portanto, o presente estudo teve como objetivo avaliar as características das equipes de TxR em Minas Gerais, o estado com o segundo maior número de TxR no Brasil, explorando as características de formação e práticas.

## Métodos

Convidamos todos os centros de TxR ativos em Minas Gerais na época da concepção do estudo (2021)^3^ para participar deste estudo transversal. Foram considerados centros ativos aqueles que realizaram pelo menos um TxR/ano nos últimos 5 anos^3^. O convite para participar e o formulário de coleta de dados foram enviados por e-mail aos responsáveis pelos centros ativos, baseados no Registro Brasileiro de Transplantes^3^. Após o aceite, o convite e os formulários de coleta de dados foram enviados a todos os membros da equipe de TxR listados pelo coordenador do centro de TxR. Antes de responder às perguntas, os entrevistados concordaram em participar assinando eletronicamente um termo de consentimento livre e esclarecido.

O estudo foi realizado de acordo com a Declaração de Helsinque e suas emendas e foi aprovado pelo Comitê de Ética em Pesquisa do Hospital Universitário da Universidade Federal de Juiz de Fora (registro: CAAE48030521.9.0000.5133; número de aprovação: 4.825.883).

Coletamos dados demográficos e sobre as características dos centros de transplante, composições das equipes, formas de remuneração e formação profissional dos cirurgiões de cada equipe.

Os seguintes dados foram coletados para os centros: localização (capital ou outras regiões); tipo de hospital (instituição de ensino ou outro); composição de especialistas das equipes de transplante (urologista, cirurgião vascular, cirurgião geral ou outros); número de especialistas exclusivos na equipe de TxR; atividade no processo de doação de órgãos; transplante pediátrico; percentual de transplantes pagos pelo Sistema Único de Saúde (SUS) (100%, 75–100%, 50–75%, 25–50%, menos de 25% e 0%); remuneração da equipe (salário mensal fixo, por produção ou outro); remuneração por disponibilidade para longa distância; e tipo de contrato da equipe com a instituição (contrato coletivo ou contrato público formal)^12,17^.

Para os cirurgiões de TxR, coletamos dados sobre idade, sexo e raça. Quanto à formação profissional, foram avaliados o tempo desde a graduação em medicina (anos), a especialidade cirúrgica (urologia, cirurgia vascular ou cirurgia geral), o tipo de formação de pós-graduação em transplante (residência ou especialização), o tempo decorrido desde a formação em transplante (anos), o tempo como parte da equipe de TxR (anos), o tipo de atividade na área de TxR (transplante e doação de rim) e a participação em mais de uma equipe de TxR^12,17^.

Os dados descritivos foram relatados como média (± desvio padrão), mediana (mínimo e máximo) e proporção, de acordo com o tipo de variável. A normalidade das variáveis contínuas foi avaliada pelo teste de Kolmogorov-Smirnov e as comparações foram realizadas por meio do teste t de Student ou do teste U de Mann-Whitney, conforme a distribuição das variáveis. As variáveis categóricas foram comparadas utilizando o teste qui-quadrado ou o teste exato de Fisher. Para comparar os cirurgiões de TxR de acordo com os níveis de treinamento específico, avaliamos a associação entre as variáveis exploratórias usando modelos de regressão linear e logística para variáveis quantitativas e categóricas. A significância estatística foi estabelecida em p < 0,05 e todos os dados foram analisados com o uso do Stata (Versão 14, StataCorp, College Station, TX, EUA).

## Resultados

Dos 19 centros de TxR ativos em Minas Gerais, 17 (89%) concordaram em participar e 50% dos cirurgiões, informados pelo representante do centro, responderam à pesquisa (Figura 1). Quarenta e sete por cento estavam localizados na capital, 88% eram instituições de ensino e em 94% o SUS custeava mais de 75% dos TxR realizados. O número de cirurgiões foi de 6 ± 2,4 por equipe, composta principalmente por urologistas (3,5 ± 1,9 por equipe). Com relação às formas de remuneração, 76,5% dos cirurgiões recebiam uma remuneração com base na produção e 76,5% não possuíam contratos formais de trabalho com o hospital (Tabela 1).

**Figura 1:** Fluxo da amostra. TxR – transplante renal.

**Tabela 1:** Características dos 17 centros ativos de TxR estudados

Variável	% / Média ± DP
Localizado na capital	47% (8/17)
Hospital de ensino	89% (15/17)
Número de cirurgiões na equipe	6 ± 2,4
Urologista	3,47 ± 1,94
Cirurgião vascular	1,65 ± 1,66
Cirurgião geral	0 (0–2)*
Composição da equipe de TxR	
Urologista	94,2% (16/17)
Cirurgião vascular	64,7% (11/17)
Cirurgião geral	29,4% (5/17)
Cirurgião transplantador	17,6% (3/17)
Envolvido exclusivamente com TxR	5,9% (1/17)
Doação de órgãos	94,1% (16/17)
Número de cirurgiões na doação de órgãos	3,65 ± 2,26
Transplante pediátrico	47% (8/17)
Pagamento pelo Sistema Único de Saúde	
100%	70,6% (12/17)
75% ou mais	23,5% (4/17)
Menos de 25%	5,9% (1/17)
Remuneração por produção	76,5% (13/17)
Remuneração por chamadas à distância	17,6% (3/17)
Contratos de trabalho	
Grupo de cirurgiões	76,5% (13/17)
Público formal	23,5% (4/17)

TxR: transplante renal, DP: desvio padrão.

*mediana e intervalo interquartil.

Os cirurgiões de TxR eram, em sua maioria, homens (95%) com uma média de idade de 46,3 ± 9,7 anos. A maioria dos cirurgiões realizou sua residência médica em áreas cirúrgicas. O tempo médio desde a formação em TxR foi de 13 ± 9,4 anos e o tempo médio de atuação em equipes de TxR foi de 10 ± 9,7 anos. A maioria dos cirurgiões de TxR estava envolvida tanto no TxR quanto na doação de órgãos. Somente um cirurgião trabalhava exclusivamente na área de TxR, e 13% dos cirurgiões faziam parte de mais de uma equipe. Apenas 25,6% dos participantes haviam concluído uma residência ou especialização em transplante, e somente um havia concluído uma residência formal em TxR (Tabela 2). Entre os cirurgiões que não realizaram especialização ou residência médica em TxR, os motivos mais citados foram a falta de programas (48%), o fato de julgarem desnecessário (26%) e a dificuldade em conciliar com o trabalho (19%) (Figura 2). Os cirurgiões com formação específica em transplante apresentaram características demográficas e de prática profissional semelhantes, exceto pelo fato de serem mais propensos a receber pagamentos com base na produção por não terem contratos formais com hospitais (Tabela 3).

**Tabela 2:** Características demográficas, educacionais e práticas dos cirurgiões que atuam em TxR

Variável	% / Média ± DP
Sexo (masculino)	95% (37/39)
Caucasiano	92,3% (36/39)
Idade (anos)	46,3 ± 9,7
Tempo desde a graduação (anos)	21,6 ± 10
Especialidade cirúrgica	
Urologia	59% (23/39)
Cirurgia geral	20,5% (08/39)
Cirurgia vascular	17,9% (07/39)
Transplante renal	2,6% (01/39)
Tipo de programa de especialização	
Residência médica em área especializada	74,4% (29/39)
*Especialização* em cirurgia de transplante	20,5% (08/39)
*Especialização* em cirurgia de TxR	2,6% (01/39)
Residência médica em cirurgia de TxR	2,6% (01/39)
Especialização ou residência em transplante ou TxR	25,6% (10/39)
Tempo desde a formação em transplante (anos)	13 ± 9,4
Tempo de atuação em equipes de TxR (anos)	10 ± 9,7
Áreas de atividade em equipes de TxR	
Transplantes e doação de órgãos	69,2% (27/39)
Somente transplante	20,5% (08/39)
Somente doação	10,3% (04/39)
Participação em mais de uma equipe	13% (05/39)
Envolvido exclusivamente em TxR	0

TxR: transplante renal, DP: desvio padrão.

**Figura 2:** Razões para a falta de formação específica em transplante renal.

**Tabela 3:** Comparações entre características demográficas e práticas de cirurgiões com e sem formação específica em transplante ou TxR

Variável	Com formação específica (n = 10)	Sem formação específica (n = 29)	OR (IC 95%); valor de p
***Demografia e formação***		
Idade (anos)	44,2 ± 10,9	46,9 ± 9,4	1,03 (0,95–1,12); p = 0,756
Sexo masculino	90% (9/10)	96,5% (28/29)	2,8 (0,16–49,21); p = 0,418
Caucasiano	90% (9/10)	93,1% (27/29)	1,22 (0,36–4,09); p= 0,166
Tempo desde a graduação em medicina (anos)	19,8 ± 11,2	22,2 ± 18,6	1,03 (0,95–1,11); p = 0,710
Tempo desde a formação em TxR (anos)	13 ± 9,13	13 ± 9,72	1,00 (0,93–1,08); p = 0,504
***Perfil profissional***		
Hospital universitário	90% (9/10)	96,5% (28/29)	0,34 (0,02–6,81); p = 0,418
Tempo em TxR (anos)	8,5 ± 6,75	10,5 ± 10,6	1,04 (0,95–1,13); p = 0,757
Número de cirurgiões na equipe	5,9 ± 2,4	7,1 ± 2,3	1,24 (0,91–1,68); p = 0,898
Realiza TxR e doação de órgãos	80% (8/10)	65% (19/29)	1,46 (0,44–4,82); p = 0,622
Número de equipes de TxR	1,2 ± 0,42	1,1 ± 0,31	0,52 (0,07–3,86); p = 0,259
Equipe paga 100% pelo SUS	40% (4/10)	69% (20/29)	0,30 (0,07–1,38); p = 0,104
Remuneração por produção	90% (9/10)	48,3% (14/29)	6,66 (1,72–25); p = 0,021
Contrato de trabalho formal	10% (1/10)	51,7% (15/29)	0,15 (0,04–0,58); p = 0,021

TxR: transplante renal, DP: desvio padrão, OR: *odds ratio*, IC 95%: intervalo de confiança de 95%, SUS: Sistema Único de Saúde.

## Discussão

Nessa análise realizada em Minas Gerais, a maioria dos 17 centros de TxR estava localizada em hospitais de ensino, a maioria dos TxR foi custeada pelo Sistema Único de Saúde e a maioria dos profissionais era remunerada por produção e não possuía nenhum contrato formal. Os 39 cirurgiões avaliados eram majoritariamente do sexo masculino, com mais de 20 anos de formação; a maioria era urologista e não possuía formação específica em TxR (residência ou especialização) devido à falta de oportunidade ou disponibilidade. Este foi um estudo transversal de base populacional que, pela primeira vez, segundo nosso conhecimento, avaliou as características de formação e atividade dos cirurgiões que realizam TxR no Brasil. Embora nossa amostra apresente características específicas do Brasil, trata-se de um dos países com alta atividade de transplantes, e nossos desafios podem ser semelhantes aos de outros países que compartilham essas características.

Constatamos que os centros estavam predominantemente localizados na capital e em hospitais de ensino, o que revela a complexidade do TxR como procedimento. Achados semelhantes foram relatados por Ximenes et al.^17^ no único outro estudo brasileiro que avaliou 20 equipes atuantes no estado de São Paulo, o estado com o maior número de TxR do país^2,3^. A distribuição dos centros de TxR em áreas com melhores recursos econômicos^9,18^ e instalações altamente estruturadas, como hospitais de ensino, já foi descrita anteriormente^19^. O principal responsável pelo pagamento dos procedimentos de TxR foi o sistema público de saúde, o que amplia o acesso ao tratamento, uma característica dos países com elevada prevalência de TxR^18,20^. Encontramos uma porcentagem maior de procedimentos financiados com recursos públicos (mais de 75% dos procedimentos) em comparação com o outro estudo brasileiro, 94% vs. 55%^16^. Por outro lado, a remuneração pela disponibilidade para longa distância é maior em São Paulo^17^. Entretanto, independentemente dessas pequenas diferenças, o cenário de trabalho está longe de ser ideal, principalmente devido à fragilidade das condições de trabalho em contratos temporários.

Mais de 90% dos cirurgiões de TxR eram do sexo masculino, semelhante a relatos anteriores^21,22^. Pela primeira vez, foram explorados os detalhes da formação profissional dos cirurgiões brasileiros de TxR. A maioria dos profissionais recebeu treinamento em TxR durante a residência cirúrgica, mas somente um quarto passou por treinamento específico em TxR por meio de um programa de residência médica ou *especialização*. Esse percentual é inferior ao relatado por Florence et al.^22^ em uma amostra dos Estados Unidos (EUA). Eles estudaram 171 cirurgiões atuando em TxR e relataram que 95% haviam concluído a formação especializada em transplante. Essa diferença também se reflete no tempo total de formação, que foi 2,6 anos mais curto em nossa amostra^22^. Atualmente, após o estabelecimento de uma legislação com normas detalhadas e conteúdo educacional, os EUA contam com 66 programas de formação em transplantes, a maioria deles multiorgãos, e 205 programas de TxR^23,24^. Em alguns países, como a Alemanha, não há regulamentação para a formação médica em transplante de órgãos, e não há necessidade de certificação de especialização na área para a prática de transplante de órgãos sólidos. Foi proposta a certificação por uma associação médica na disciplina de cirurgia^11^. Dois estudos alemães relataram baixos níveis de formação específica em transplantes, com apenas 17% e 11,8% dos cirurgiões transplantadores possuindo qualquer tipo de certificação^10,13^.

Atualmente, no Brasil, falta uma proposta universal e bem definida para a formação em TxR, embora existam programas de treinamento especializado acreditados para essa área. As regulamentações brasileiras para a composição de equipes de TxR para acreditação pelo Ministério da Saúde^16^ estipulam que a equipe deve ser composta por dois urologistas ou um urologista e um cirurgião geral com residência ou título de especialista, com pelo menos seis meses de treinamento formal em TxR em um hospital universitário ou de excelência na área^16^. Entretanto, diferentemente dos EUA^12^, no Brasil não há detalhes sobre como o programa de capacitação deve ser implementado e, por exemplo, não há um número mínimo de procedimentos exigidos que sejam assistidos ou realizados sob supervisão^25^. Alguns países definiram o número mínimo de procedimentos e exames de proficiência como um requisito para a acreditação como parte das equipes de cirurgia de TxR^10,26^. Após a criação das regulamentações brasileiras para residência médica em transplante em 2010^25^, houve um crescimento nos programas de residência médica em TxR e especializações no país. Atualmente, existem 11 centros com treinamento cirúrgico exclusivo em TxR, nove em urologia e dois em cirurgia vascular (comunicação pessoal, Comitê Nacional Brasileiro de Residência Médica, 2020). Assim, os caminhos atuais para a formação em TxR no Brasil são um programa específico de residência médica, um curso de pós-graduação *latto sensu* (programa de especialização) ou o exercício profissional de um especialista em urologia ou cirurgia geral em um centro credenciado para TxR. Embora o número de programas especializados esteja crescendo, a maioria dos cirurgiões de TxR em nosso estudo relatou a indisponibilidade de programas de treinamento como o motivo para não concluir esse tipo de educação complementar e necessária. Não há informações disponíveis sobre o preenchimento de vagas de nível inicial nesse programa especializado em TxR.

O tempo médio de trabalho em equipes de TxR foi menor em nossa pesquisa do que o relatado pelo outro estudo brasileiro, possivelmente porque a carreira em TxR é nova, altamente especializada e menos atrativa, conforme relatado^14,27^. Um achado importante de nosso estudo foi que os cirurgiões com formação específica em transplante tinham contratos institucionais e formas de remuneração menos favoráveis. Isso pode ocorrer porque a formação específica em TxR só foi disponibilizada recentemente; portanto, os cirurgiões que realizaram essa formação são mais jovens e ainda estão em início de carreira. Outro aspecto relevante é que, mesmo com a formação mais adequada, o campo de trabalho ainda não favorece esses profissionais, uma vez que a regulamentação não exige formação específica em TxR^12,13^.

Atualmente, a cirurgia de transplante não é reconhecida como uma especialidade pelo Conselho Federal de Medicina e pela Associação Médica Brasileira (AMB)^28^. Isso dificulta o processo de seleção para esse tipo de profissional, pois a legislação não permite a abertura de vagas para especialidades não incluídas na lista da AMB. Consequentemente, é difícil formar novas equipes ou expandir equipes de TxR ativas em entidades públicas, como é o caso de muitos centros de TxR no Brasil^2^. O baixo índice de profissionais com formação específica em TxR nos centros e as formas mais precárias de remuneração e contratos que encontramos refletem as dificuldades acima mencionadas e reforçam o impacto da falta de regulamentações específicas.

Este estudo apresentou algumas limitações, principalmente por se tratar de um estudo baseado em questionários. Embora a taxa de resposta dos responsáveis pelos centros de TxR em Minas Gerais tenha sido elevada (89%), a taxa de resposta dos cirurgiões dos centros participantes foi de 50%, o que pode ter causado um viés nos resultados. No entanto, a porcentagem de participação foi semelhante à de relatos anteriores^12,22^. Apesar de nosso estudo ter se baseado em dados de um estado brasileiro, ele fornece um esboço inicial das ações necessárias para melhorar a formação em TxR e destaca a necessidade de dados nacionais mais detalhados. Outro aspecto que precisa ser aprofundado é a potencial melhoria do desempenho em centros com profissionais com formação específica em TxR como parte de suas equipes.

Em conclusão, constatamos que os cirurgiões que atuavam nos centros de TxR em Minas Gerais eram, em sua maioria, do sexo masculino, urologistas, envolvidos na captação e transplante de órgãos e sem formação específica em TxR por falta de oportunidade ou disponibilidade. A maioria dos centros de TxR eram instituições de ensino que recebiam remuneração por produção e por meio do sistema público de saúde. Uma minoria dos cirurgiões possuía residência ou *especialização* em transplante e trabalhava sob contratos menos favoráveis. Acreditamos que esses dados podem ajudar as associações médicas e instituições governamentais a elaborar políticas para aumentar o número de cirurgiões especializados em TxR e equipes qualificadas para, em última instância, aumentar o número de TxR.

## Data Availability

Os conjuntos de dados gerados e/ou analisados durante o estudo atual estão disponíveis com o autor correspondente, mediante solicitação.
